# Attention Deficit/Hyperactivity Disorder and Increased Engagement in Sexual Risk-Taking Behavior: The Role of Benefit Perception

**DOI:** 10.3389/fpsyg.2019.01043

**Published:** 2019-05-22

**Authors:** Tali Spiegel, Yehuda Pollak

**Affiliations:** The Seymour Fox School of Education, The Hebrew University of Jerusalem, Jerusalem, Israel

**Keywords:** attention deficit/hyperactivity disorder, adults, sexual risk-taking behavior, psychological risk–return model, risk and benefit perception

## Abstract

Attention deficit/hyperactivity disorder (ADHD) has been linked to higher engagement in sexual risk-taking behavior (SRTB). The current study aims to establish the link between ADHD symptoms and SRTB in the general population and to examine whether an exaggerated perceived benefit of the positive outcomes of SRTB explains that link. A scale for measuring the frequency, likelihood, perceived benefit, and perceived risk of SRTB was developed. Young adult sexually active participants who did not have a stable partnership completed the above scale, as well as a scale of ADHD symptoms. The level of ADHD symptoms positively correlated with the frequency and likelihood of SRTB, even when the overall level of sexual behavior was controlled for. ADHD symptoms also correlated with the perceived benefit of SRTB, but not with the perceived risk of SRTB. Mediation analysis confirmed an indirect pathway: ADHD symptoms predicted perceived benefit of SRTB, which in turn predicted increased likelihood to engage in SRTB. These findings suggest a positive link between ADHD symptoms and SRTB in the general population, which is accounted for by an exaggerated perceived benefit of SRTB.

## Introduction

Attention deficit/hyperactivity disorder (ADHD) is a neurodevelopmental disorder characterized by inattention and/or hyperactivity/impulsivity and is associated with poor academic, social, occupational, financial, and health-related functioning (Faraone et al., 2015). People with ADHD tend to engage in risk-taking behaviors, defined as behaviors associated with a higher probability of undesirable outcomes (Boyer, 2006), such as significant physical injuries or financial loss. These risk-taking behaviors include, among others, substance use, smoking, reckless driving, and sexual risk-taking behavior (SRTB) (Nigg, 2013).

Sexual risk-taking behavior may result in a variety of undesirable outcomes, including family conflicts, financial loss, and damaged reputation, but the two most commonly addressed concerns are unintended pregnancies and sexually transmitted diseases, such as HIV/AIDS (Turchik and Garske, 2009). SRTB involves having sex at an early age, having unprotected sex, and having multiple sexual partners (Martinez et al., 2011). These health-threatening behaviors have been linked to drug and alcohol abuse, psychopathology, early parenthood, educational problems, and convictions (Viner, 2005).

A small number of studies have focused on the link between ADHD and SRTB. Prospective studies showed that childhood ADHD is associated with earlier sexual activity, a higher number of sexual partners, more sex outside of a relationship, more sexually transmitted diseases, more partner pregnancies, and teenage parenthood (Barkley et al., 2006a; Flory et al., 2006; Hosain et al., 2012; Sarver et al., 2014; Ostergaard et al., 2017).

A relevant question is what may explain the link between ADHD and SRTB. Individual differences associated with both ADHD and SRTB may have the potential to act as mediators. Lifetime conduct disorder symptoms, which are closely related to ADHD, were found to contribute to the link between ADHD and SRTB significantly (Barkley et al., 2006b; Ramos Olazagasti et al., 2013; Sarver et al., 2014). In another study, ADHD made a unique contribution to STRB above and beyond conduct problems (Flory et al., 2006).

Excessive SRTB may also result from higher engagement in sexual behavior in general, as extra sexual activity, when not in the context of a stable, monogamous relationship, increases the possibility of exposure to risky sexual intercourse. In the literature regarding ADHD and risk taking in driving, the measured level is usually controlled against the total exposure/driving time of the participant (Knouse et al., 2005; Vaa, 2014). However, to the best of our knowledge, the studies concerning the link between ADHD and SRTB were performed without control against the total engagement in sexual behavior.

The present study suggests observing SRTB through the prism of Weber’s behavioral decision theory (Weber et al., 2002). According to this version of a psychological risk–return theory, a choice to engage in risk-taking behavior should be analyzed in terms of benefit and risk, as they are subjectively perceived by the decision maker. Weber contends that people differ in the perceived level of risk and benefit they ascribe to the behavior. In addition, though to a lesser extent, they differ in the attitudes toward these perceptions (namely in the weight the perceived risk and benefit have on the choice to engage in the behavior). Individual differences in benefit and risk perceptions and attitudes lead to different levels of engagement in risk-taking behavior, i.e., higher benefit perception and attitude and/or lower risk perception and attitude account for the choice to engage in risk-taking behavior. A recent study examined whether the link between ADHD symptoms and the overall level of risk-taking behavior is explained by differences in benefit and risk perceptions and attitudes. The study found a positive correlation between ADHD symptoms and the level of benefit perception. Furthermore, a mediation analysis revealed an indirect pathway between ADHD and risk-taking behavior through increased benefit perception, suggesting that people with ADHD engage in risk-taking behavior more often than controls, since they view the benefits of engaging in these risky behaviors as greater (Shoham et al., 2016).

Therefore, the goal of the study presented here was to examine the association between ADHD and SRTB and the influence of the perceived benefit on this association. In order to measure SRTB, a questionnaire consisting of 15 risky sexual behaviors was assembled. For each behavior, the participants were asked to rate the frequency, likelihood, perceived benefit, and perceived risk of engagement in that behavior. The frequency section contained an additional item probing for the frequency of engagement in “sexual activity of any kind” (not only risky) to control for individual differences in general sexual activity.

In this paper, we favored a dimensional approach for the conceptualization of ADHD and, thus, referred to ADHD as a continuous trait, based on mental health dimensional models (Coghill and Sonuga-Barke, 2012). According to the dimensional approach, people with ADHD represent the end of a continuum of the level of ADHD symptoms’ distribution, rather than a distinct clinical category (Levy et al., 1997). In line with this approach, the current study measured ADHD continuously based on symptoms level.

Hence, the present study hypotheses were as follows: (1) ADHD symptoms in the general population would be positively associated with the engagement in SRTB, even after controlling for general sexual activity; (2) an indirect link between ADHD and SRTB would be found through the link between ADHD and benefit perception.

## Materials and Methods

### Participants

The experiment was approved by the ethics committee of the Seymour Fox School of Education, at the Hebrew University. One-hundred thirty adults, aged 19–39, participated in the study, of which 10 participants were excluded for excessive missing values or contradictory responses (see below in the “Results” section). All participants were sexually active (had at least one intercourse over the past 6 months) and were not in a stable relationship. The participants were recruited through advertisements in the social media “Facebook,” by different experimenters and from two separated locations (Tel-Aviv and Jerusalem districts).

### Protocol and Measurement

All participants filled out an online informed consent form and were given the opportunity to participate in a lottery to win 500 NIS (∼125 euros) by leaving an email address. The following questionnaires were completed by the participants:

#### Demographic Questionnaire

The participants provided background information on age, gender, education, religiousness level, and sexual orientation.

#### The Sexual Risk-Taking Behavior Scale (SRTB)

The Sexual Risk-Taking Behavior Scale (SRTB) (see Appendix 1) consists of 15 risky sexual activities (such as, “anal sex without the use of a condom,” “casual sex”). Inspired by Weber’s Domain-Specific Risk-Taking (DOSPERT) scale assessment (Blais and Weber, 2006), the SRTB measures the likelihood and frequency of engaging in and the perceived benefits and risks of each of the risky sexual behaviors using seven-point Likert scales for likelihood and perception and an eight-point Likert scale for frequency (likelihood: 1 = extremely unlikely, 7 = extremely likely; benefit perception: 1 = no benefits, 7 = great benefits; risk perception: 1 = not at all risky, 7 = extremely risky; frequency: 1 = never, 8 = at least once a day). The frequency scale measured SRTB over the past year. In addition, in order to measure general sexual activity, respondents were asked to indicate the frequency they participated in “sexual activity of any kind.” Construct validity was assessed via correlations between likelihood and frequency measurements and between likelihood and perception measurements. Additional validation was obtained by comparing the risk perception scores of items that self-evidently differed in the risk level (see the “Results” section).

#### The Adult ADHD Self-Report Scale (ASRS-V1.1) (Kessler et al., 2005)

The Adult ADHD Self-Report Scale (ASRS-V1.1) (Kessler et al., 2005) is a dimensional measure of ADHD symptoms. It includes 18 items (9 items of inattention and 9 items of hyperactivity/impulsivity) corresponding to the *Diagnostic and Statistical Manual of Mental Disorder, 4th ed.* diagnostic criteria of ADHD, each measured for its frequency on a Likert scale ranging from 1 (never) to 5 (very often). The questionnaire has high internal consistency (α = 0.88). The scale’s sensitivity and specificity are 68.4 and 99.6%, respectively (Kessler et al., 2005; Adler et al., 2006). The Hebrew version has high test–retest reliability estimates (*p* = 0.6–0.9), high internal consistency of α = 0.89, and sensitivity and specificity of 62.7 and 68%, respectively (Zohar and Konfortes, 2010).

#### Sexual History and ADHD Diagnosis Questionnaire

In order to characterize the sample in terms of sexual history, participants provided information on sexual debut, lifetime number of sexual partners, history of sexually transmitted diseases, and HIV tests. In addition, participants reported on any history of a diagnosis of ADHD and the use of medications to treat ADHD.

### Statistical Analysis

Total scores were calculated for each participant on each questionnaire. Using Weber’s regression equation (Weber et al., 2002), for each subject, we regressed the likelihood of engagement in SRTB on the perceived benefit and perceived risk and calculated the coefficients that index the individual attitudes toward the perceived benefit and risk. Skewness and kurtosis tests were conducted to examine the normality of the distribution of the variables. For further covariation, we examined whether there were any statistically significant associations between demographic variables and the study variables.

To test the first hypothesis, a regression analysis of the relations between ADHD symptoms and frequency of SRTB was computed while controlling for the general sexual activity variable. To test the second hypothesis, direct and indirect effects of ADHD symptoms on the likelihood of SRTB were calculated using the multiple mediation approach and SPSS macro (PROCESS, Model 6) provided by Hayes (2013). The significance of effects was tested via bootstrap analysis (5,000 samples), which is commonly performed in multiple mediator analyses, given its advantage of greater statistical power without assuming multivariate normality in the sampling distribution, assuming only the sample is representative of the population. Statistical mediation is demonstrated via a significant indirect effect (i.e., if the 95% bias-corrected confidence interval for the parameter estimate does not contain zero). All analyses were conducted using SPSS 25.0 including an SPSS macro designed for assessing multiple mediation models.

## Results

### Preliminary Analyses

Data from participants, which met the following criteria, were not included in the analyses: (1) missing values exceeded 30% in one or more of the scales (six participants). (2) Responses contradicted each other (e.g., frequency of risky sexual behavior exceeded the frequency of sexual behavior in general). When one contradiction was observed, only the contradicting items were removed from the database. When more than one contradiction was observed, the entire data of the participant were removed (four participants).

### Sample Characteristics

Participants were recruited from two locations: Tel Aviv and Jerusalem districts (*n* = 33 and 87, respectively). Mean age of the sample was 25.77 (±4.33). The sample consisted of 72.5% females; 90.8% of the participants had high education; 89.2% of the sample identified as non-religious; and 92.5% identified as heterosexual. Regarding diagnosis of and treatment for ADHD, 19.2% reported that they had been formally diagnosed with ADHD, and 14.2% of the sample reported ever using medication for ADHD. Regarding history of risky sexual behavior, 70.8% reported having three or more partners so far, 45.0% have ever been screened for STD with 5.0% of the sample found positive, 41.7% of the participants (or their partners) have ever used “morning after” pills, and 5.0% had unintended pregnancy.

### Reliability and Validity of the Measurements

Internal consistency indices of the likelihood, frequency, and perception scales were in the acceptable to good range (Cronbach’s alpha 0.78–0.88). Skewness and kurtosis tests were used to confirm the normality of distribution. Skewness indices of all scales, except for the frequency scale, were in the −1 to 1 range (0.19, 0.35, −0.05, −0.19, 0.95, and 1.34 for likelihood, benefit perception, benefit attitude, risk perception, risk attitude, and frequency, respectively). Kurtosis indices of all scales, except for the benefit attitude, risk attitude, and frequency scales, were in the −1 to 1 range (0.05, 0.35, 2.20, 0.01, 3.17, and 2.00 for likelihood, benefit perception, benefit attitude, risk perception, risk attitude, and frequency, respectively). As three out of six scales were not normally distributed, further analyses were conducted using non-parametric tests.

Several analyses were conducted to support the trustworthiness of the responses and the validity of the scales. First, we compared ASRS mean scores between the participants who reported having and not having an ADHD diagnosis. As expected, the ASRS scores of the participants with a history of an ADHD diagnosis were significantly higher [*M* = 3.14, *SD* = 0.70, and *M* = 2.53, *SD* = 0.46, respectively, *t*(26.74) = 3.98, *P* < 0.001, Hedges’ *g* = 1.19]. Next, we analyzed the responses to specific items in the likelihood, frequency, and risk perception scales, under the assumption that their associated level of risk differs in an obvious way. Items 9a, b and 9c, d in the likelihood and risk perception levels (and the corresponding items 11a, b and 11c, d in the frequency scale) refer to sex with a new partner and differ in whether the sexual history of the partner is known. As expected, the likelihood and frequency scores were lower, and the risk perception score was higher when the history of the partner was unknown (*P* < 0.001). Similarly, items 10a, b and 10c, d in the likelihood and risk perception levels (and the corresponding items 12a, b and 12c, d in the frequency scale) refer to regular but non-committed sex and differ in whether the sexual history of the partner is known. As expected, the likelihood and frequency scores were lower, and the risk perception score was higher when the history of the partner was unknown (*P* < 0.001).

Median and 25–75% values of the ASRS and SRTB scores are presented in Table 1. A positive Spearman rho correlation was found between the likelihood and frequency scales (*r* = 0.63, *P* < 0.001). A regression analysis was conducted with the SRTB likelihood score as the predicted variable, and both perception and attitude score as predictors. This model accounted for 50% of the variance in the SRTB likelihood score. As expected, the benefit perception and attitude scores positively predicted SRTB likelihood, whereas the risk perception and attitude scores negatively predicted SRTB likelihood. A bootstrap analysis revealed that benefit and risk perceptions scores, as well as the risk attitude score, contributed to the prediction of SRTB likelihood above and beyond all other variables (β = 0.59, −0.21, and −0.18, respectively, 95% CIs did not include zero).

**Table 1:** Descriptive statistics of ASRS and SRTB scales.

	Median	25–75%
ASRS (mean item score)	2.58	2.28–3.06
Inattention	2.67	2.33–3.00
Hyperactivity	2.56	2.22–3.00
SRTB scales		
Frequency of engagement	1.63	1.34–1.94
Likelihood of engagement	3.06	2.35–3.62
Benefit perception	2.88	2.31–3.54
Risk perception	4.84	4.06–5.31
Perceived-benefit attitude	0.60	0.30–0.88
Perceived-risk attitude	−0.30	−0.50–−0.10

*N = 120 (87 females, 33 males); ASRS, Adult ADHD Self-Report Scale; SRTB, sexual risk-taking behavior.*

### Confounds

The correlations between the variables included in the hypotheses and the demographic variables were examined. As presented in Table 2, gender, age, religiousness, and sample location, but not level of education, were significantly related to one or more of the SRTB variables and were further controlled for in later analyses. Of the various demographic variables, gender was related to SRTB in the most consistent manner. Female participants reported lower engagement in SRTB and lower benefit perception, as well as higher risk perception and risk aversion. As gender was related to SRTB scores in a consistent manner, an exploratory analysis was conducted to examine whether gender and ADHD interact in predicting SRTB. Moderation analyses failed to show any significant interaction between ADHD and gender in predicting the frequency, likelihood, benefit, and risk perception, as well as perceived benefit and risk attitudes [*F*(1,112) = 0.051, 0.019, 0.477, 0.665, 0.205, and 0.001, respectively, *p*≥ 0.412, age, religiousness, sexual orientation, and sample location covariated].

**Table 2:** Spearman rho correlation between demographic variables and SRTB scales.

	Sample location	Gender	Age	Religiousness	Education	Sexual orientation
Frequency of engagement	−0.237^∗∗^	−0.195^∗^	0.228^∗^	0.138	−0.029	0.259^∗^
Likelihood of engagement	0.076	−0.231^∗^	0.080	−0.267^∗∗^	−0.006	0.153
Benefit perception	−0.100	−0.219^∗^	0.112	−0.154	−0.017	0.123
Risk perception	−0.141	0.197^∗^	0.071	0.195^∗^	0.059	−0.152
Perceived-benefit attitude	0.105	0.133	−0.138	0.077	0.102	−0.211^∗^
Perceived-risk attitude	−0.060	0.210^∗^	−0.202^∗^	0.126	−0.014	−0.022

*SRTB, sexual risk-taking behavior; sample location was coded as 1 = Tel-Aviv district, 2 = Jerusalem district; gender was coded as 1 = male, 2 = female; religiousness was coded as 1 = secular, 2 = traditional, 3 = Orthodox; sexual orientation was coded as 1 = heterosexual, 2 = not heterosexual. ^∗^p < 0.05, ^∗∗^p < 0.01.*

### Hypotheses Testing

To test the first hypothesis, hierarchical linear regression was conducted for analyzing the contribution of ADHD symptoms to the prediction of the frequency of engagement in SRTB. Gender, age, religiousness, sexual orientation, sample location, as well as the frequency of general sexual activity were entered in the first block, and the ASRS score was entered in a subsequent block. The first block explained 44.6% of the variance in the SRTB score, whereas the ASRS explained an additional 3.4% of the variance, indicating a small effect size (*f*^2^ = 0.035). Male gender, general sexual activity, and ASRS score predicted SRTB above and beyond all other predictors (see Table 3). Re-analyzing the same model, this time with inattention and hyperactivity symptoms separately entered into the second block, elicited similar results, with neither symptom cluster predicting SRTB above and beyond the other cluster.

**Table 3:** Prediction of sexual risk-taking behavior.

Predictor	SRTB		
	Δ*R*^2^	*B*	95% CI
**Step 1**			

	44.6		
Age		0.00	−0.03–0.02
Gender		−0.21^∗^	−0.40–−0.03
Religiousness		−0.06	−0.17–0.05
Sexual orientation		0.21	−0.03–0.64
Sample location		−0.18	−0.44–0.10
General sexual activity		0.22^∗^	0.15–0.28

**Step 2**			

	3.4		
Age		0.00	−0.02–0.03
Gender		−0.24^∗^	−0.42–0.05
Religiousness		−0.06	−0.15–0.05
Sexual orientation		0.13	−0.07–0.49
Sample location		−0.17	−0.42–0.10
General sexual activity		0.21^∗^	0.15–0.27
ASRS		0.19^∗^	0.04-0.34

*ASRS, Adult ADHD Self-Report Scale; SRTB, sexual risk-taking behavior. ^∗^95% confidence interval does not contain zero.*

Mediation analyses were conducted to test the second hypothesis. The ASRS score served as a predictor, the risk and benefit perceptions and attitudes as mediators, the likelihood scores as the predicted variable, and gender, age, religiousness, sexual orientation, sample location, as well as the frequency of general sexual activity as covariates.

**FIGURE 1:**
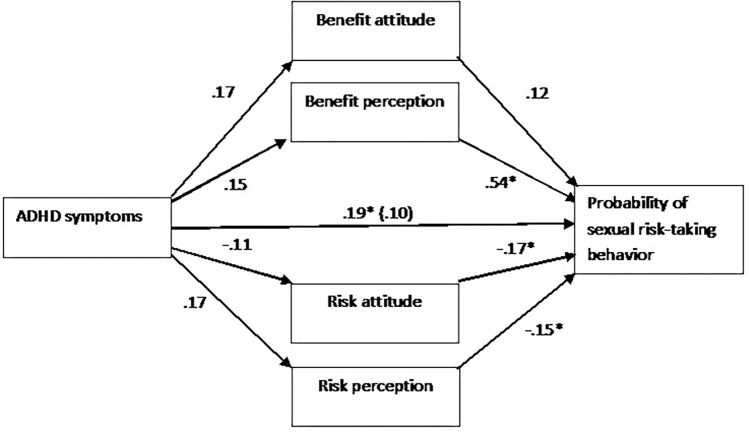
Path analysis portraying the direct and indirect pathways predicting the likelihood of sexual risk-taking behavior (SRTB). Values reflect standardized regression coefficients of the indirect effects and of the direct effect (after considering other mediators) of Adult ADHD Self-Report Scale (ASRS) on SRTB. The significance of effects was tested via a 5,000-sample bootstrap analysis (the effect is considered significant if the 95% bias-corrected confidence interval for the parameter estimate does not contain zero). The covariates of age, gender, religiousness, sexual orientation, sample location, and the frequency of general engagement in sexual behavior are not shown for visual clarity. *N* = 120 (87 females, 33 males). ^∗^95% confidence interval does not contain zero.

The path analysis in Figure 1 depicts the direct effects and indirect pathways of the model. Together, the model accounted for 54.6% of the variance in SRTB (*P*< 0.001). The standardized regression coefficient between ADHD symptoms and SRTB before considering mediators, and between ADHD symptoms and benefit perception, was statistically significant. The bootstrapped standardized indirect effect mediated by benefit perception was significant and of moderate effect size. The indirect effects of ADHD symptoms, mediated by risk perception, risk attitude, and benefit attitude, were not significant. ADHD symptoms did not predict SRTB after accounting for the indirect effect (see Table 4 for coefficients and CIs).

**Table 4:** The direct and indirect pathways of the link between the ASRS score and the likelihood of engagement in SRTB.

	Model *R*^2^	Indirect effect				Direct effect
		Benefit perception	Risk perception	Perceived-benefit attitude	Perceived-risk attitude	
ASRS	54.6	0.081^∗^	0.017	0.020	−0.030	0.102
		95% CI [0.007, 0.171]	95% CI [−0.010, 0.063]	95% CI [−0.009, −0.064]	95% CI [−0.084, 0.009]	95% CI [−0.039, 0.243]

*ASRS, Adult ADHD Self Report Scale; SRTB, sexual risk-taking behavior. ^∗^95% confidence interval does not contain zero.*

## Discussion

The study focused on the relationship between ADHD symptoms, engagement in SRTB, and perception and attitudes regarding the outcomes of SRTB. The results of the current study confirmed the following hypotheses: (a) the level of ADHD symptoms in the general population correlates with the frequency of engagement in SRTB even after controlling for general engagement in sexual behavior; (b) a link between ADHD and SRTB exists through the positive correlation of ADHD and benefit perception.

### ADHD and Increased SRTB

Self-reported ADHD symptoms predicted the self-reported real-life frequency and the hypothetical likelihood of engagement in SRTB. These findings are in agreement with other studies documenting increased SRTB by people with ADHD (Barkley et al., 2006a; Flory et al., 2006), as well as with studies reporting correlation between the level of ADHD symptoms and SRTB in the general population (Sarver et al., 2014; Marsh et al., 2015; Isaksson et al., 2018).

The link between ADHD and increased engagement in SRTB may be explained by increased overall engagement in sexual behavior. To test this hypothesis, the reported frequency of the general sexual activity was used as a covariate. ADHD symptoms still predicted SRTB even after covariating for the general sexual activity.

Importantly, the link between ADHD and risk-taking behavior is not limited to SRTB. Numerous studies consistently reported associations between ADHD and higher engagement in other risk-taking behaviors, e.g., substance use, reckless driving, and gambling (for review, see Nigg, 2013), with scales measuring overall levels of risk-taking behavior (Pollak et al., 2016; Shoham et al., 2016; Pollak et al., 2017), as well as with increased risk taking on laboratory tasks (Mowinckel et al., 2015; Dekkers et al., 2016).

### ADHD Symptoms and SRTB-Related Risk/Benefit Perceptions and Attitudes

According to the behavioral decision theory (Weber et al., 2002), a psychological risk–return model, risk and return are subjectively evaluated. According to this model, SRTB in ADHD cases may be explained by a less negative/more positive attitude, or weight given to risk and return, respectively, but also by differences in subjective evaluation of risk and return, i.e., risk and benefit perception. We used Weber’s behavioral decision theory to operationalize risk and benefit perception and attitude. Perceptions were measured directly by asking participants to rate the magnitude of risk and benefit they ascribe to different SRTBs, whereas attitudes were calculated by regressing the likelihood of engagement in SRTB on perceptions.

A main finding of the current study is that ADHD symptoms correlate with the perception of the benefits associated with SRTB. Mediation analysis supported a model, according to which the link between ADHD symptoms and SRTB is indirect through the link between ADHD and higher benefit perception. The indirect pathways, through benefit attitude, risk perception, and risk attitude, were not significant. These findings are in accord with a recent study demonstrating that ADHD symptoms correlate with increased benefit perception of the positive outcomes of widespread risk-taking behavior, but not with the risk perception of the negative outcomes of risk-taking behavior (Shoham et al., 2016). Importantly, given the cross-sectional nature of this study, it should be highlighted that the supported mediation model is only statistical. Further research may use a longitudinal design, which enables examining whether the link between ADHD and SRTB is stable across time and whether there is evidence for temporal precedence, which are important conditions of causality.

### Clinical Implications

The investigation of the mechanisms underlying SRTB among people with ADHD has important clinical implications. Specifically, it informs prescriptive research with the goal of helping people with ADHD to optimize their sexually related decision-making and counter their engagement in dangerous sexual activities. Our findings suggest that interventions aimed at reducing SRTB in adults should include measures of their ADHD symptoms as well as their perceptions of the benefits (and risks) of engaging in SRTB. Interventions may be devised considering the research, which would deal with external regulation and strategies that consider the individuals’ perceptions.

### Limitations

This study has several limitations: The sample size was not big enough to further examine factors that affect the link between ADHD and SRTB. The convenience sampling resulted in an over-representation of women and of individuals with higher education and with a history of ADHD diagnosis. However, the degree of education did not correlate with risk measures. In addition, engagement in SRTB was assessed using self-report, which was not validated by a collateral report.

## Ethics Statement

This study was carried out in accordance with the recommendations of the Declaration of Helsinki with written informed consent from all subjects. All subjects gave written informed consent in accordance with the Declaration of Helsinki. The protocol was approved by the ethics committee of the Seymour Fox School of Education, at The Hebrew University of Jerusalem.

## Author Contributions

TS and YP designed the study and analyzed the data. TS collected the data and wrote the manuscript. YP reviewed and revised the manuscript.

## Conflict of Interest Statement

The authors declare that the research was conducted in the absence of any commercial or financial relationships that could be construed as a potential conflict of interest.
