# Corrigendum: Attention Deficit/Hyperactivity Disorder and Increased Engagement in Sexual Risk-Taking Behavior: The Role of Benefit Perception

**DOI:** 10.3389/fpsyg.2019.02152

**Published:** 2019-09-19

**Authors:** Tali Spiegel, Yehuda Pollak

**Affiliations:** The Seymour Fox School of Education, The Hebrew University of Jerusalem, Jerusalem, Israel

**Keywords:** attention deficit/hyperactivity disorder, adults, sexual risk-taking behavior, psychological risk–return model, risk and benefit perception

An author name was incorrectly spelled as Yehdua Pollak. The correct spelling is Yehuda Pollak.

The authors apologize for this error and state that this does not change the scientific conclusions of the article in any way. The original article has been updated.

