# Supplementary material for: Attention Deficit/Hyperactivity Disorder and Increased Engagement in Sexual Risk-Taking Behavior: The Role of Benefit Perception
Source: Front Psychol. 2019 May 22;10:1043. doi: 10.3389/fpsyg.2019.01043 (PMC6538875; doi:10.3389/fpsyg.2019.01043)
Supplement: Supplementary file 1 [file Table_1.docx]

Appendix

Demographic questionnaire:

If you find discomfort with any of the following questions you are free to decline to answer it.

Thank you for your cooperation

Fill in the following details:

Gender: F / M

Age:_____

Place of birth:_____________

Immigration date:_ _/_ _/

Religious affiliation: not religious / traditional / religious / ultra-orthodox / other________

Years of education:

High school education

Bachelor’s degree (also during)

Master's degree (also during)

Ph.D. (also during)

other______________

sexual orientation: heterosexual / homosexual / other___________

**Sexual risk-taking behavior questionnaire –** likelihood**:**

Your participation in this survey is anonymous and part of a research study. You may refuse to take part in the study or leave the study at any time. You may answer only part of the questions.

For each of the following statements, please indicate the likelihood that you would engage in the described activity or behavior, if you were to find yourself in that situation. Provide a rating from "*Extremely Unlikely*" to "*Extremely Likely*", using the following scale:

| **7** | **6** | **5** | **4** | **3** | **2** | **1** |
| --- | --- | --- | --- | --- | --- | --- |
| **Extremely likely** | **Moderately likely** | **Somewhat Likely** | **Not Sure** | **Somewhat Unlikely** | **Moderately Unlikely** | **Extremely Unlikely** |

| 1 2 3 4 5 6 7 | Oral sex | 1 |
| --- | --- | --- |
| 1 2 3 4 5 6 7 | Casual sexual intercourse | 2 |
| 1 2 3 4 5 6 7 | Having sex with multiple partners | 3 |
| 1 2 3 4 5 6 7 | Dangerous masturbation (such as, auto-erotic suffixation) | 4 |
| 1 2 3 4 5 6 7 | Anal sex without a condom | 5 |
| 1 2 3 4 5 6 7 | Sexual activity with multiple participants | 6 |
| 1 2 3 4 5 6 7 | Sexual activity involving violence which may lead to significant injury | 7 |
| 1 2 3 4 5 6 7 | Sex under influence of substances (drugs/alcohol) | 8 |
| 1 2 3 4 5 6 7 | Sex with a new partner: | 9 |
| 1 2 3 4 5 6 7 | 1. when his/her STD history is unknown and without using protection of any kind. |  |
| 1 2 3 4 5 6 7 | 1. when his/her STD history is unknown, without using a condom, yet using birth control |  |
| 1 2 3 4 5 6 7 | 1. when you know he/she doesn’t have history of STD and without using protection of any kind. |  |
| 1 2 3 4 5 6 7 | 1. when you know he/she doesn’t have history of STD, without using a condom, yet using birth control |  |
| 1 2 3 4 5 6 7 | Uncommitted yet stable sexual relationship | 10 |
| 1 2 3 4 5 6 7 | 1. when his/her STD history is unknown and without using protection of any kind. |  |
| 1 2 3 4 5 6 7 | 1. when his/her STD history is unknown, without using a condom, yet using birth control |  |
| 1 2 3 4 5 6 7 | 1. when you know he/she doesn’t have history of STD and without using protection of any kind. |  |
| 1 2 3 4 5 6 7 | 1. when you know he/she doesn’t have history of STD, without using a condom, yet using birth control |  |

**Sexual risk-taking behavior questionnaire –** **Risk Perception**:

Your participation in this survey is anonymous and part of a research study. You may refuse to take part in the study or leave the study at any time. You may answer only part of the questions.

For each of the following statements, please indicate how risky you perceive each situation. Provide a rating from *"Not at all Risky"* to *"Extremely Risky"*, using the following scale:

| Extremely risky | Very Risky | Risky | Moderately risky | Somewhat Risky | Slightly Risky | Not at all Risky |
| --- | --- | --- | --- | --- | --- | --- |
| 7 | 6 | 5 | 4 | 3 | 2 | 1 |

| 1 2 3 4 5 6 7 | Oral sex | 1 |
| --- | --- | --- |
| 1 2 3 4 5 6 7 | Casual sexual intercourse | 2 |
| 1 2 3 4 5 6 7 | Having sex with multiple partners | 3 |
| 1 2 3 4 5 6 7 | Dangerous masturbation (such as, auto-erotic suffixation) | 4 |
| 1 2 3 4 5 6 7 | Anal sex without a condom | 5 |
| 1 2 3 4 5 6 7 | Sexual activity with multiple participants | 6 |
| 1 2 3 4 5 6 7 | Sexual activity involving violence which may lead to significant injury | 7 |
| 1 2 3 4 5 6 7 | Sex under influence of substances (drugs/alcohol) | 8 |
| 1 2 3 4 5 6 7 | Sex with a new partner: | 9 |
| 1 2 3 4 5 6 7 | 1. when his/her STD history is unknown and without using protection of any kind. |  |
| 1 2 3 4 5 6 7 | 1. when his/her STD history is unknown, without using a condom, yet using birth control |  |
| 1 2 3 4 5 6 7 | 1. when you know he/she doesn’t have history of STD and without using protection of any kind. |  |
| 1 2 3 4 5 6 7 | 1. when you know he/she doesn’t have history of STD, without using a condom, yet using birth control |  |
| 1 2 3 4 5 6 7 | Uncommitted yet stable sexual relationship | 10 |
| 1 2 3 4 5 6 7 | 1. when his/her STD history is unknown and without using protection of any kind. |  |
| 1 2 3 4 5 6 7 | 1. when his/her STD history is unknown, without using a condom, yet using birth control |  |
| 1 2 3 4 5 6 7 | 1. when you know he/she doesn’t have history of STD and without using protection of any kind. |  |
| 1 2 3 4 5 6 7 | 1. when you know he/she doesn’t have history of STD, without using a condom, yet using birth control |  |

**Sexual risk-taking behavior questionnaire –** **Benefit Perception**:

Your participation in this survey is anonymous and part of a research study. You may refuse to take part in the study or leave the study at any time. You may answer only part of the questions.

For each of the following statements, please indicate how beneficial you perceive each situation. Provide a rating from *"No benefits at all"* to *"Great benefits"*, using the following scale:

| **Great benefits** |  |  | **Moderate benefits** |  |  | **No benefits at all** |
| --- | --- | --- | --- | --- | --- | --- |
| **7** | **6** | **5** | **4** | **3** | **2** | **1** |

| 1 2 3 4 5 6 7 | Oral sex | 1 |
| --- | --- | --- |
| 1 2 3 4 5 6 7 | Casual sexual intercourse | 2 |
| 1 2 3 4 5 6 7 | Having sex with multiple partners | 3 |
| 1 2 3 4 5 6 7 | Dangerous masturbation (such as, auto-erotic suffixation) | 4 |
| 1 2 3 4 5 6 7 | Anal sex without a condom | 5 |
| 1 2 3 4 5 6 7 | Sexual activity with multiple participants | 6 |
| 1 2 3 4 5 6 7 | Sexual activity involving violence which may lead to significant injury | 7 |
| 1 2 3 4 5 6 7 | Sex under influence of substances (drugs/alcohol) | 8 |
| 1 2 3 4 5 6 7 | Sex with a new partner: | 9 |
| 1 2 3 4 5 6 7 | 1. when his/her STD history is unknown and without using protection of any kind. |  |
| 1 2 3 4 5 6 7 | 1. when his/her STD history is unknown, without using a condom, yet using birth control |  |
| 1 2 3 4 5 6 7 | 1. when you know he/she doesn’t have history of STD and without using protection of any kind. |  |
| 1 2 3 4 5 6 7 | 1. when you know he/she doesn’t have history of STD, without using a condom, yet using birth control |  |
| 1 2 3 4 5 6 7 | Uncommitted yet stable sexual relationship | 10 |
| 1 2 3 4 5 6 7 | 1. when his/her STD history is unknown and without using protection of any kind. |  |
| 1 2 3 4 5 6 7 | 1. when his/her STD history is unknown, without using a condom, yet using birth control |  |
| 1 2 3 4 5 6 7 | 1. when you know he/she doesn’t have history of STD and without using protection of any kind. |  |
| 1 2 3 4 5 6 7 | 1. when you know he/she doesn’t have history of STD, without using a condom, yet using birth control |  |

**Sexual risk-taking behavior questionnaire –** **Frequency**:

**Instructions to Frequency scale for participants**

Your participation in this survey is anonymous and part of a research study. You may refuse to take part in the study or leave the study at any time. You may answer only part of the questions.

For each of the following statements, please indicate the frequency you participate in these activities/behaviors. Provide a rating from *"Never"* to *"At least once a day"*, using the following scale:

| **At least once a day** | **Almost every day** | **Numerous times a week** | **Once in a week-two weeks** | **Once a month** | **Once in two-three months** | **Seldom** | **Never** |
| --- | --- | --- | --- | --- | --- | --- | --- |
| **8** | **7** | **6** | **5** | **4** | **3** | **2** | **1** |

| 1 2 3 4 5 6 7 8 | Sexual activity of any kind | 1 |
| --- | --- | --- |
| 1 2 3 4 5 6 7 8 | masturbation | 2 |
| 1 2 3 4 5 6 7 8 | Oral sex | 3 |
| 1 2 3 4 5 6 7 8 | Casual sexual intercourse | 4 |
| 1 2 3 4 5 6 7 8 | Having sex with multiple partners | 5 |
| 1 2 3 4 5 6 7 8 | Dangerous masturbation (such as, auto-erotic suffixation) | 6 |
| 1 2 3 4 5 6 7 8 | Anal sex without a condom | 7 |
| 1 2 3 4 5 6 7 8 | Sexual activity with multiple participants | 8 |
| 1 2 3 4 5 6 7 8 | Sexual activity involving violence which may lead to significant injury | 9 |
| 1 2 3 4 5 6 7 8 | Sex under influence of substances (drugs/alcohol) | 10 |
| 1 2 3 4 5 6 7 8 | Sex with a new partner: | 11 |
| 1 2 3 4 5 6 7 8 | 1. when his/her STD history is unknown and without using protection of any kind. |  |
| 1 2 3 4 5 6 7 8 | 1. when his/her STD history is unknown, without using a condom, yet using birth control |  |
| 1 2 3 4 5 6 7 8 | 1. when you know he/she doesn’t have history of STD and without using protection of any kind. |  |
| 1 2 3 4 5 6 7 8 | 1. when you know he/she doesn’t have history of STD, without using a condom, yet using birth control |  |
| 1 2 3 4 5 6 7 8 | Uncommitted yet stable sexual relationship | 12 |
| 1 2 3 4 5 6 7 8 | 1. when his/her STD history is unknown and without using protection of any kind. |  |
| 1 2 3 4 5 6 7 8 | 1. when his/her STD history is unknown, without using a condom, yet using birth control |  |
| 1 2 3 4 5 6 7 8 | 1. when you know he/she doesn’t have history of STD and without using protection of any kind. |  |
| 1 2 3 4 5 6 7 8 | 1. when you know he/she doesn’t have history of STD, without using a condom, yet using birth control |  |

**Sexual history and ADHD diagnosis questionnaire**

Your participation in this survey is anonymous and part of a research study. You may refuse to take part in the study or leave the study at any time. You may answer only part of the questions.

Please answer the following questions:

Your age at the start of sexual activity: _____

Did you have more than three sexual partners? Yes / No

Have you been tested for HIV or sexually transmitted diseases? Yes / No

If your answer is yes, how many times? _____

Have you ever been infected with STD / HIV? Yes / No

Have you / your partner ever used "morning after" pills? Yes / No

If your answer is yes, how many times? _____

Have you ever had an unwanted pregnancy / got a sexual partner into unwanted pregnancy? Yes / No

Have you been ever diagnosed with ADHD? Yes / No

Have you ever used medication used to treat ADHD (e.g., Ritalin)? Yes / No
